# Magnitude of Thrombocytopenia and Associated Factors among Pregnant Women Attending the Antenatal Care Service Unit of Wachemo University Nigist Ellen Mohammed Comprehensive Specialized Hospital Hosanna, Southern Ethiopia

**DOI:** 10.1155/2024/8163447

**Published:** 2024-04-16

**Authors:** Dembelo Tirago, Tilahun Yemane, Edosa Tadasa

**Affiliations:** ^1^Department of Medical Laboratory Science, Hossana Health Science College, Hosanna, Ethiopia; ^2^School of Medical Laboratory Sciences, Faculty of Health Sciences, Institute of Health, Jimma University, Jimma, Ethiopia

## Abstract

**Background:**

Thrombocytopenia is the second most common hematological disorder in pregnancy after anemia worldwide and affects 7-8% of all pregnancies. Pregnant women with thrombocytopenia have complications of excessive bleeding during or after childbirth, exudation at the cesarean section, and neonatal thrombocytopenia. Therefore, the main aim of this study was to assess the magnitude of thrombocytopenia and its associated factors among pregnant women attending the Antenatal Care Service Unit of Wachemo University Nigist Ellen Mohammed Comprehensive Specialized Hospital Hosanna, Southern Ethiopia.

**Materials and Methods:**

A cross-sectional study was conducted from June 1 to August 30, 2022, involving 209 consecutive pregnant women who came to the hospital during the study period for antenatal care follow-up. Sociodemographic, reproductive, and other clinical data were collected using a structured questionnaire. A four-milliliter venous blood specimen was collected for complete blood analysis and peripheral blood smear. The data were analyzed by using SPSS version 25. Descriptive statistical analysis and bivariate and multivariate logistic regression analyses were performed. A *P* value ≤0.05 was considered to indicate statistical significance.

**Results:**

The overall magnitude of thrombocytopenia among pregnant women was 14.8% (95% CI: 10, 19.6). The prevalence of mild, moderate, and severe thrombocytopenia was 77.4%, 16.1%, and 6.5%, respectively. Multivariate logistic regression revealed that rural residence (AOR = 2.6 and 95% CI = 1.02, 7.12), cigarette smoking (AOR = 8.4 and 95% CI = 1.86, 38), anemia (AOR = 8.3 and 95% CI = 2.7, 25.6), and alcohol consumption (AOR = 8.2 and 95% CI = 2.17–31) were significantly independent factors associated with the development of thrombocytopenia.

**Conclusion:**

The magnitude of thrombocytopenia in this study was 14.8%. Rural residence, cigarette smoking, alcohol consumption, and anemia were significantly associated with thrombocytopenia. Therefore, the platelet count should be routinely determined during antenatal care visits for proper diagnosis and to minimize bleeding during and/or after childbirth.

## 1. Introduction

Thrombocytopenia is a common hematologic abnormality and is defined as a platelet count less than 150 × 10^9^/L. However, many consider a cutoff value of 100 × 10^9^/L to be more appropriate for identifying clinically significant thrombocytopenia [1, 2]. Thrombocytopenia is the second most common hematological abnormality during pregnancy after anemia. It occurs in 7-8% of all pregnancies [3, 4].

Platelets are anucleate cell fragments of megakaryocytes that play an important role in hemostasis. They arise from the fragmentation of megakaryocytes, bone marrow cells with high polyploidy that arise through the process of mitosis and endomitosis [5]. After leaving the marrow space, approximately one third of the platelets are sequestered in the spleen, and the remaining two thirds circulate for 7–10 days. Typically, only a small portion of the platelet mass is used in the process of hemostasis, so most platelets circulate until they age and are removed by phagocytic cells. The normal platelet count is between 150 and 450 × 10^9^/l [6].

The severity of thrombocytopenia was divided into 3 grades depending on the PLT: mild thrombocytopenia with a PLT between 100 and 150 × 10^9^/l and moderate thrombocytopenia with a PLT between 50 and 100 × 10^9^/l. Severe thrombocytopenia was defined as a level less than 50 × 10^9^/L [5]. The American Society of Hematology requires that treatment be initiated if the PLT is less than 30 × 10^9^/L or if bleeding occurs between the 2nd and 3rd trimesters of pregnancy [4].

The PLT during pregnancy varies depending on the trimester and gradually decreases from the first trimester until delivery. The physiological causes of thrombocytopenia include an increase in plasma volume (dilution effect), accumulation of platelets in the spleen due to spleen enlargement during pregnancy, and accumulation of platelets in the intervillous space of the placenta [7, 8].

Thrombocytopenia can be caused by decreased bone marrow production, increased splenic sequestration, or accelerated platelet destruction. During pregnancy, most cases are due to increased destruction of platelets, which may be caused by the immune system, abnormal activation of platelets, or consumption of platelets due to exposure to abnormal vessels or excessive bleeding. Reduced platelet production during pregnancy occurs less frequently and is usually associated with bone marrow disorders or nutritional deficiencies [1, 6]. Pregnant women with low platelet counts tend to be much less symptomatic due to the procoagulant state caused by elevated levels of factor VIII, fibrinogen, and von Willebrand factor (VWF); suppressed fibrinolysis; and reduced protein S activity [9].

Thrombocytopenia during pregnancy can have various causes. Some of these associations are pregnancy specific, while others can occur outside of pregnancy. These include gestational thrombocytopenia, hypertensive disorders of pregnancy and hemolysis, elevated liver enzymes and low platelet count syndrome (HELLP), liver disease (acute fatty liver of pregnancy), drug-induced thrombocytopenia, immune thrombocytopenic purpura, virus-induced thrombocytopenia, disseminated intravascular coagulation (DIC), hemolytic uremic syndrome (HUS), vitamin B12 and folic acid deficiency, and myelopthisis [10, 11].

Immune thrombocytopenic purpura (ITP) is caused by platelet autoantibodies against various glycoprotein complexes of the platelet membrane and is responsible for approximately 5% of the cases of thrombocytopenia during pregnancy [12]. Pregnant women with hypertension account for 5–21% of the cases of maternal thrombocytopenia [13]. Preeclampsia (PE) and HELLP syndrome are considered to be the cause of thrombocytopenia during pregnancy in approximately 14.2% of the cases [12]. All pregnant women with a PLT less than 100 × 10^9^/L require detailed hematological and obstetric consultation to rule out more serious conditions [14]. In sub-Saharan Africa, the prevalence is slightly higher at 15.3%. This condition is responsible for up to 10% of the cases of postpartum hemorrhage in developing countries, with a maternal mortality rate of 5.26% [15, 16].

Hematologic changes during pregnancy are common and can result in maternal and fetal morbidity [7]. Approximately 8–10% of pregnant women, especially in the third trimester, are affected by thrombocytopenia and 75% of these cases are due to the benign process of gestational thrombocytopenia [17]. It is often associated with an increased risk of bleeding before and after birth, particularly during cesarean section or other surgical procedures during pregnancy [4, 18].

The most common cause of gestational thrombocytopenia (GT) is thrombocytopenia during pregnancy, which commonly occurs in 5–11% of pregnant women [19]. It is typically an incidental diagnosis, and the pathogenesis of this disease is unclear [20].

Thrombocytopenia during pregnancy results in additional, often invasive and unnecessary, examinations and a cesarean section. Pregnant women with thrombocytopenia experience complications such as excessive bleeding during or after delivery, cesarean section discharge, stillbirths, and neonatal thrombocytopenia. Examination and treatment of this condition can be expensive, painful, and potentially undesirable for the patient [18]. In Ethiopia, particularly in selected study area complete blood counts are routinely performed for all pregnant women. However, there is no follow-up on platelet count during third trimester, labour, and delivery until severe complications were ensured. Determining magnitude of thrombocytopenia severity and associated factors plays a great role in the management of thrombocytopenic women and is important to develop evidence-based intervention measures, which are not well known in this study area. Therefore, the aim of this study was to evaluate the magnitude of thrombocytopenia and its associated factors during pregnancy at Nigist Ellen Mohammed Memorial Comprehensive Specialized Hospital, Wachemo University.

## 2. Materials and Methods

### 2.1. Study Design, Area, and Period

A hospital-based cross-sectional study was conducted from June 1 to August 30, 2022, at Wachemo University Nigist Ellen Mohammed Memorial Comprehensive Referral Hospital (WUNEMMCSH), which is located in Hosanna Town, Hadiya Zone, Southern Nations Nationalities, and People's Region (SNNPR). The town is 232 km from the capital city of Addis Ababa to the southwest and 157 km from the regional city of Hawassa. The town lies on average 2,177 m above the sea level. This hospital was established in the 1976 EC, and its catchment area population is estimated to be approximately 3,476,386. On average, 1244 pregnant women attended the hospital annually. The study was conducted in an antenatal care clinic.

### 2.2. Study Population

All pregnant women aged between 15 and 49 years who attended the WUNEMCSH antenatal care service clinic during the study period and met the inclusion criteria were included. Study participants who took medications such as heparin and nonsteroidal anti-inflammatory drugs such as aspirin and had bleeding disorders, splenomegaly, or chronic illness (HCV, HBV, or hypertension) were excluded from the study after reviewing their medical records.

### 2.3. Sample Size Determination and the Sampling Technique

The sample size was calculated by using the single population proportion formula by taking the 14.5% prevalence of thrombocytopenia [21] and by considering a 95% confidence interval (CI) and 5% margin of error. The final sample size after adding a 10% nonresponse rate was 209. All pregnant women who attended the WUNEMCSH antenatal care service clinic during the study period were consecutively included in the study until attaining the final sample size.

### 2.4. Data Collection Methods and Instruments

Data on sociodemographic characteristics and clinical and other related factors were collected by trained data collectors. After the purpose of the study was explained, a laboratory technologist collected 4 mL of venous blood from each study participant into tubes containing ethylenediaminetetraacetic acid (EDTA) for complete blood count analysis and blood smear preparation. Platelet counts, platelet parameters, and other hematological parameters were measured using a MINDRAY BC-3000 PLUS automated hematology analyzer (Shenzhen Mindray Bio-Medical Electronics Co., Ltd., China). Thin blood smears were prepared from thrombocytopenic specimens, air-dried, labeled, and stained with Wright's stain to assess platelet morphology (small platelets and giant platelets) and the presence of malaria parasites. In addition, platelet aggregation was observed to distinguish between pseudo thrombocytopenia and true thrombocytopenia.

Thrombocytopenia is said to be present when the platelet count of the pregnant women is less than 150 × 10^9^/L. The PLT count between 100 and 150 × 10^9^/l is considered mild thrombocytopenia, levels ranging between 50 and 100 × 10^9^/l are considered as moderate thrombocytopenia, and levels less than 50 × 10^9^/L are considered as severe thrombocytopenia [22].

### 2.5. Data Quality Control

The quality of the data was guaranteed by implementing quality control (QC) measures throughout the whole process of the laboratory work, data collection, data entry, and data processing. The quality of the data was maintained by pretesting the questionnaire before the actual data collection. MINDRAY BC-3000 PLUS hematology analyzer was checked for linearity and validity of the results by analyzing whole-blood quality control material (high, normal, and low). The quality of the blood specimens was maintained during sample collection, transportation, and processing by mixing an appropriate volume of blood and anticoagulant following the standard operating procedure at each step and analyzing the samples within time. All reagents were checked for their expiration date and prepared according to the manufacturer's instructions. Wright stains were filtered every day by using filter paper and stored in locked cabinets away from moisture and sunlight. In addition, thin blood films prepared from thrombocytopenic clients and stained with Wright stain were examined to confirm the presence of platelet aggregation, which was used to differentiate true thrombocytopenia from pseudothrombocytopenia.

### 2.6. Data Processing and Analysis

The data collected for the study were cleaned, entered into Epi Data version 4.6, and analyzed using the Statistical Package for Social Science (SPSS) version 25 (IBM Corporation, Chicago, USA). Descriptive statistics, frequency tables, graphs, and logistic regression were used to describe the study variables and to determine the impact of the independent variables on the prevalence of thrombocytopenia. Bivariate analysis was performed to select candidate variables for multivariate analysis, and variables with a *P* value <0.25 were selected as candidates. Multivariate logistic regression analysis was used to identify risk factors associated with thrombocytopenia, 95% confidence intervals were calculated, and a *P* value of 0.05 or less was used to indicate statistical significance.

### 2.7. Ethical Clearance

Ethical clearance was obtained from the Institutional Review Board (IRB) of the Institute of Health, Jimma University (letter number IRB000843/2022; date, April 30/2022). Permission to conduct the study was obtained from the Wachemo University Nigist Ellen Mohammed Comprehensive Specialized Hospital. All study participants were made aware of the study's purpose, risks, and benefits, and their written informed consent was obtained from each participant before they took part in the study per the Declaration of Helsinki and universal Good Clinical and Laboratory Practice (GCP and GCLP) principles. Patient confidentiality, equity of services, and patient interests were ensured during the study period. All data collected during the study were treated with strict confidentiality and used only for this study.

## 3. Results

### 3.1. Sociodemographic Characteristics of the Study Participants

A total of 209 pregnant women were included in this study with a response rate of 100%. The mean (±SD) age of the study participants was 26.1 ± 4.983 years (age ranged from 17 to 43). About 61.2% (*n* = 128) of the study participants were urban area residents. The majority (85.6%; *n* = 179) of the participants were married. Among the study participants, about 33.5% (*n* = 70) were in primary school, and 56.5% (*n* = 118) were housewives (Table 1).

### 3.2. Reproductive, Clinical, and Other Characteristics of the Study Participants

The mean (±SD) gestational age of the study participants was 26.94 ± 7.63 weeks (gestational age ranged from 8 to 41). The majority (52.6%, *n* = 110) of the study participants were in the third trimester. About 49.8% (*n* = 104) of the study participants had previously undergone ANC follow-up. Among the study participants, about 24.4% (*n* = 51) had a history of heavy menstrual bleeding, and 14.4% (*n* = 30) had hemoglobin levels less than 11 g/dl (Table 2).

### 3.3. Magnitude and Severity Pattern of Thrombocytopenia among Study Participants

**Figure 1:**
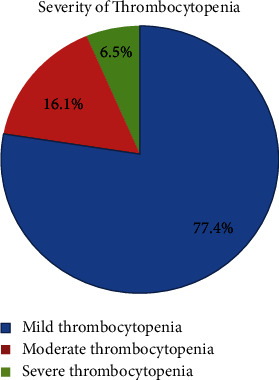
Severity pattern of thrombocytopenia among pregnant women at Wachemo University Nigist Ellen Mohammed Comprehensive Specialized Hospital Hosanna, Southern Ethiopia, from June to August 30, 2022.

The mean (±SD) platelet count among the study participants was 234.1 ± 96.1 × 10^9^/L. The overall magnitude of thrombocytopenia among the study participants was 31 (14.8%), with a 95% CI of 10 (19.6). Among thrombocytopenic study participants, 24 (77.4%), 5 (16.1%), and 2 (6.5%) had mild, moderate, and severe thrombocytopenia, respectively (Figure 1).

Among the study participants, the proportion of thrombocytopenia was 19.9% (*n* = 16) in rural residences, 15.7% (*n* = 11) in primary school, 33.3% (*n* = 6) in smoking cigarettes, 50% (*n* = 11) in alcohol consumption, 46.7% (*n* = 14) among hemoglobin level less than 11 g/dl, 48.4% (*n* = 15) within gestational age of third trimester, and 64.5% (*n* = 20) among study participant within the age group of 25–34 years. Among those with a history of malaria infection, 24% had thrombocytopenia and 33.3% had thrombocytopenia among smoking cigarette participants (Table 3).

Among the study participants, 12% (*n* = 1), 16.5% (*n* = 15), and 13.6% (*n* = 15) of the participants had thrombocytopenia in different trimesters/gestational ages, first trimester, second trimester, and third trimester, respectively, with 95% CI (10, 19.6) (Table 3).

Thin blood films were prepared from thrombocytopenic clients, and Wright staining was used to confirm the presence of platelet aggregation, satelitism, and the presence of the malaria parasite. The commonest blood picture indicates that there was no hemiparasite, platelet aggregation, or platelet satelitism in the blood film.

### 3.4. Factors Associated with Thrombocytopenia in the Study Participants

According to our bivariate analysis, rural residence, educational status, previous ANC follow-up, history of malaria, eating meat or animal products, smoking cigarette habit, alcohol consumption, hemoglobin level, and middle upper arm circumference (MUAC) were identified as candidate variables associated with thrombocytopenia multivariate analysis by considering *P* value <0.25 (Table 3).

### 3.5. Multivariate Logistic Regression Analysis of Factors Associated with Thrombocytopenia

The variable included in the final logistic regression analysis was rural residence; smoking, alcohol consumption, and hemoglobin level were significantly associated with thrombocytopenia (Table 4).

The odds of being were thrombocytopenic approximately 3 times higher among rural residents than among urban residents (AOR = 2.6, 95% CI = 1.02, 7.12, and *P*=0.045). The adjusted odds ratio (OR) revealed that pregnant women who smoked cigarettes were 8 times more likely to be thrombocytopenic than their counterparts (AOR = 8.4, 95% CI = 1.86, 38, and *P*=0.006), being anemic increase the likelihood of thrombocytopenia by eight times greater than that of nonanemic pregnant women (AOR = 8.3, 95% CI = 2.7, 25.6, and *P* < 0.01). A significantly greater prevalence of thrombocytopenia was observed in alcohol consumer participants (50%) than in nonalcohol consumer participants (AOR = 8.2, 95% CI = 2.17–31, *P*=0.002).

## 4. Discussion

Thrombocytopenia during pregnancy affects about 1 in 10 pregnant women worldwide. The rate of thrombocytopenia in pregnant women is 4 times greater than that in nonpregnant women. Therefore, timely diagnostic, preventive, and therapeutic measures are necessary for the effective management of thrombocytopenia during pregnancy [23]. Thrombocytopenia is the second most common hematologic disorder in the world and affects 7–10% of all pregnancies. This condition is responsible for up to 10% of postpartum hemorrhages in developing countries, for which the maternal mortality rate is 5.26% [15, 16].

The present study attempted to evaluate the magnitude of thrombocytopenia, severity patterns, and associated factors in pregnant women at Wachemo University Nigist Ellen Mohammed Memorial Compressive Specialized Hospital in southern Ethiopia. Our findings showed a 14.8% (95% CI: 10, 19.6) overall magnitude of thrombocytopenia among pregnant women; out of them, 24 (77.4%), 5 (16.1%), and 2 (6.5%) had mild, moderate, and severe thrombocytopenia, respectively.

Getawa et al. reported that the overall pooled prevalence of thrombocytopenia among pregnant women in Africa was 10.23% (CI = 7.44–13.02) [24]. The overall prevalence of thrombocytopenia obtained in our study was consistent with studies done in Ethiopia 14.5% [21], 13.5% [3], and 10.2% [25], India 10.2% [26], Libiya 18% [27], Ghana 15.3% [5], Nigeria 13.5% [28], and Uganda 15.8% [15].

The present study showed a greater incidence of thrombocytopenia than the studies conducted in Ethiopia (7.7–9.9%) [17, 18, 29], India (8.8%) [30], Libya (8.3%) [31], and Baghdad (7.1%) [32]. The difference in the prevalence of thrombocytopenia might be due to differences in sociodemographics, sample size, variability of the automated analyzer, geographical variation, or lifestyle.

A systematic review and meta-analysis of global thrombocytopenia showed that the prevalence of thrombocytopenia is four times greater in pregnant women than in nonpregnant women [23]; the average global prevalence was 8.4% (CI: 6.9–10.1%) [23]. However, our study finding was higher than the global prevalence of thrombocytopenia. Therefore, these findings provide assurance or confirmation that routine screening and follow-up programs are needed to identify pregnant women with thrombocytopenia and offer them essential interventions.

Hematological changes during pregnancy are common and can lead to maternal and fetal morbidity, especially in the third trimester, when the condition is affected by thrombocytopenia. There are many causes of thrombocytopenia during pregnancy, ranging from mild to life-threatening, and thrombocytopenia needs to be diagnosed and treated promptly [7, 17]. The severity patterns among the thrombocytopenic pregnant women in our study were 77.4%, 16.1%, and 6.5% in mild, moderate, and severe thrombocytopenia, respectively. These findings are consistent with studies conducted in Ethiopia, Libya, India, Uganda, and Ghana [3, 15, 19, 26, 31, 33, 34], which indicate a high frequency of mild thrombocytopenia.

Several studies have reported that the prevalence of thrombocytopenia is significantly affected by different sociodemographic characteristics [18, 21, 29]. In the present study, thrombocytopenia was significantly associated with rural residence. These findings are in agreement with those of studies performed in Ethiopia [33, 34] and Libya [31]. This might be related to a lack of information about adequate nutrition, lack of information on factors causing thrombocytopenia and possible strategies to prevent risk factors, inaccessibility of health care centers to ANC follow-up, and way of life.

Alcoholism is one of the most serious socioeconomic health problems in the world [35]. According to the current study, pregnant women with a habit of alcohol consumption were 8 times more likely to develop thrombocytopenia than their counterparts were. This finding was in line with a study in Ethiopia in which the habit of alcohol consumption was more likely to lead to thrombocytopenia [21]. The possible explanations that explain these associations might be the direct toxic effect of alcohol on the suppression of blood cell production, hematopoiesis, impaired function, and a decreased platelet life span [33, 35, 36].

Cigarette smoking can induce both acute and chronic effects on platelet function. Smoking induces oxidative stress, leading to increased platelet activation and aggregation and endothelial injury [37]. The present study revealed that there was a statistically significant association between thrombocytopenia and smoking cigarettes. Smoking cigarettes were associated with 8.4 times greater odds of thrombocytopenia than nonsmoking cigarettes were. A possible explanation might be that cigarette smoke induces oxidative stress, damages endothelial tissue, and disturbs the function of platelets, especially through activation and aggregation, as well as other coagulation components, leading to thrombosis [34, 37, 38].

Anemia during pregnancy was strongly associated with thrombocytopenia in this study. The odds of having thrombocytopenia were eight times higher among women with an Hb concentration less than 11 g/dl compared to those who had hemoglobin ≥11 g/dl, which are significant (A0R: 7.7 and 95% CI: 2.8–21.6). This finding was similar to that of a study performed in Uganda and Ethiopia, where a strong association between anemia and thrombocytopenia was observed [15, 39]. Recurrent thrombocytopenia has typically been linked to iron deficiency anemia, which is prevalent throughout pregnancy. The dual role of iron in the creation of platelets, which is necessary for the production of an integral section of the platelet, and the diphasic response of platelets to erythropoietin are the hypothesized causes of thrombocytopenia in patients with IDA [40]. The findings of our study should be concluded in light of some limitations. First, the cross-sectional nature of the study design prohibited the establishment of causal links between thrombocytopenia and factors that are significantly associated with thrombocytopenia. Second, the duration of alcohol consumption per day, type of alcohol, and alcohol concentration among the study participants were not assessed. Third, data on cigarette smoking concerning the number smoked per day and the type of cigarettes smoked were not assessed among the study participants.

## 5. Conclusion

A greater magnitude of thrombocytopenia (14.8%) was observed among pregnant women. Among the thrombocytopenic pregnant women, mild thrombocytopenia was the predominant condition in our study. Rural residence, alcohol consumption, cigarette [41] smoking, and anemia were identified as independent predictors of thrombocytopenia among pregnant women. Therefore, screening should include platelet counts, especially when the woman is from a rural residence, is anemic, consumes alcohol, or is a cigarette smoker, to avoid adverse outcomes.

## Figures and Tables

**Table 1:** Sociodemographic characteristics of pregnant women attending antenatal care clinics at Wachemo University Nigist Ellen Mohammed Comprehensive Specialized Hospital Hosanna, Southern Ethiopia, from June to August 30, 2022.

Variables	Categories	Frequency	Percentage (%)
Age in year	15–24	58	27.8
	25–34	131	62.7
	≥35	20	9.6

Residence	Urban	128	61.2
	Rural	81	38.8

Marital status	Single	12	5.7
	Married	179	85.6
	Divorced	8	3.8
	Widowed	10	4.8

Educational status	Illiterate	27	12.9
	Primary school	70	33.5
	Secondary school	67	32.1
	College/University and above	45	21.5

Occupational status	Farmer	6	2.9
	Daily laborer	1	0.5
	Employee	40	19.1
	Students	29	13.9
	Merchants	15	7.2
	Housewife	118	56.5

Monthly income in ETB^*∗*^	≤1500	159	76.1
	>1500	50	23.9

ETB^*∗*^ Ethiopian birr, Employee+, governmental employee.

**Table 2:** Reproductive, clinical, and other related characteristics of pregnant women attending antenatal care clinics at Wachemo University Nigist Ellen Mohammed Comprehensive Specialized Hospital Hosanna, Southern Ethiopia, from June to August 30, 2022.

Variables	Categories	Frequency	Percentage (%)
Gestational age	First trimester	8	3.8
	Second trimester	91	43.5
	Third trimester	110	52.6

Pregnant before	Yes	130	62.2
	No	79	37.8

Number of children	No children	4	3.1
	1	22	16.9
	2	45	34.6
	3 and above	59	45.4

ANC follow-up in a previous pregnancy	Yes	104	49.8
	No	105	50.2

History of heavy menstrual bleeding	Yes	51	24.4
	No	158	75.6

History of abortion	Yes	71	34
	No	138	66

History of contraceptive usage	Yes	99	47.4
	No	110	52.6

Types of contraceptives used	Oral	26	26.3
	Depoproven	30	30.3
	Norplant	43	43.4

Iron or folic acid supplementation	Yes	107	51.2
	No	102	48.2

History of malaria	Yes	25	12
	No	184	88

Hgb level in g/dl	<11 g/dl	30	14.4
	≥11 g/dl	179	85.6

Consumption of animal products including meat	Yes	180	86.1
	No	29	13.9
How many times eat meat and animal products	Daily	3	1.7
	Every 2 day	14	14
	Every weekly	53	29.4
	Once a month	110	61.1

Eat fruit and vegetables	Yes	205	98.1
	No	4	1.9

How many times eat fruit and vegetables	Daily	16	7.8
	Every 2 day	50	24.4
	Every weekly	133	64.9
	Once a month	6	2.9

A habit of drinking coffee or tea immediately after a meal	Yes	179	85.5
	No	30	14.4

Cigarette smoking	Yes	18	8.6
	No	191	91.4

Alcohol consumption	Yes	22	10.5
	No	187	89.5

MUAC in cm	<23	114	54.5
	≥23	95	45.5

**Table 3:** Bivariate analysis of factors associated with thrombocytopenia among pregnant women attending antenatal care clinics at Wachemo University Nigist Ellen Mohammed Comprehensive Specialized Hospital Hosanna, Southern Ethiopia, from June to August 30, 2022.

Variables	Categories	Thrombocytopenia		COR 95% CI	*P* value
		No (%)	Yes (%)		
Age in year	15–24	49 (84.5)	9 (15.5%)	1	
	25–34	111 (84.4%)	20 (15.3%)	1.65 (0.32, 8.39)	0.544
	≥35	18 (90%)	2 (10%)	1.62 (0.34, 7.5)	0.537

Residence	Urban	113 (88.3%)	15 (11.7%)	1	
	Rural	65 (80.2%)	16 (19.9%)	1.85 (0.86, 3.99)	0.115

Educational status	Illiterate	24 (88.9%)	3 (11.1%)	038 (0.09, 1.53)	0.177
	Primary school	59 (84.3)	11 (15.7)	0.57 (0.22, 1.47)	0.249
	Secondary school	61 (9%)	6 (9%)	0.10 (0.10, 0.89)	0.031
	College/university and above	34 (75.6%)	11 (24.4)	1	

Monthly income in ETB^*∗*^	≤1500	137 (86.2%)	22 (13.8%)	0.732 (0.31, 1.71)	0.479
	>1500	41 (82%)	9 (18%)	1	

Gestational age in a week	First trimester	7 (87.5%)	1 (12%)	1	
	Second trimester	76 (83.5%)	15 (16.5%)	0.73 (0.10, 7.8)	0.471
	Third trimester	95 (86.4%)	15 (13.6%)	1.25 (0.57, 2.7)	0.573

Previously ANC follow-up	Yes	85 (81.7%)	19 (18.3%)	1	
	No	93 (88.6%)	12 (11.4%)	0.57 (0.26, 1.26)	0.168

History of abortion	Yes	59 (83.1%)	12 (16.9%)	1.24 (0.58, 2.79)	0.547
	No	119 (86.2)	19 (13.8%)	1	

History of contraceptive usage	Yes	85 (85.9)	14 (14.1%)	0.91 (0.41, 1.93)	0.79
	No	93 (84.5)	17 (15.5)	1	

Taken iron and folic acid supplement	Yes	92 (86%)	15 (14%)	1	
	No	86 (84.3%)	16 (15.7%)	0.87 (0.40, 1.88)	0.73

Eat meat and animal product	Yes	156 (86.7)	24 (13.3%)	1	
	No	22 (75.9%)	7 (24.1%)	2.06 (0.79, 5.36)	0.135

History of malaria	Yes	19 (76%)	6 (24%)	2.0 (0.73, 5.51)	0.176
	No	159 (86.4%)	25 (13.6%)	1	

Smoking cigarette habit	Yes	12 (66.7%)	6 (33.3%)	3.32 (1.14, 9.64)	0.027
	No	166 (86.9%)	25 (13.1%)	1	

Alcohol consumption	Yes	11 (50%)	11 (50%)	0.12 (0.46, 0.31)	<0.001
	No	167 (89.3%)	20 (10.7%)	1	

Hemoglobin level	<11 g/dl	16 (53.3%)	14 (46.7%)	8.33 (3.47, 19.98)	<0.001
	≥11 g/dl	162 (90.5%)	17 (9.5%)	1	

MUAC	<23 cm	94 (82.5%)	20 (17.5%)	1.62 (0.73, 3.58)	0.230
	≥23 cm	84 (88.4%)	11 (11.6%)	1	

(^*∗*^) Ethiopian Birr, *P* value *<*0.05 statistically significant.

**Table 4:** Multivariate logistic regressions of selected factors associated with thrombocytopenia among pregnant women attending antenatal care clinics at Wachemo University Nigist Ellen Mohammed Comprehensive Specialized Hospital Hosanna, Southern Ethiopia, from June to August 30, 2022.

Variables	Categories	Thrombocytopenia		COR (95% CI)	AOR (95% CI)	*P* value
		No (%)	Yes (%)			
Residence	Urban	113 (88.3%)	15 (11.7%)	1		
	Rural	65 (80.2%)	16 (19.9%)	1.85 (0.86, 3.9)	2.6 (1.02, 7.12)	0.045^*∗*^

Educational status	Illiterate	24 (88.9%)	3 (11.1%)	0.38 (0.09, 1.5)	0.5 (0.08, 2.99)	0.459
	Primary school	59 (84.3)	11 (15.7)	0.57 (0.22, 1.4)	0.7 (0.19, 2.71)	0.640
	Secondary school	61 (9%)	6 (9%)	0.10 (0.10, 0.8)	0.40 (0.10, 1.64)	0.207
	College/university and above	34 (75.6%)	11 (24.4)	1		

Previous ANC follow-up	Yes	85 (81.7%)	19 (18.3%)	1		
	No	93 (88.6%)	12 (11.4%)	0.57 (0.26, 1.26)	0.50 (0.19, 1.31)	0.162

Eat meat and animal product	Yes	156 (86.7)	24 (13.3%)	1		
	No	22 (75.9%)	7 (24.1%)	2.06 (0.7, 5.3)	0.34 (0.09, 1.12)	0.09

Smoking cigar rete	Yes	12 (66.7%)	6 (33.3%)	3.32 (1.1, 9.6)	8.4 (1.86, 38)	0.006^*∗*^
	No	166 (86.9%)	25 (13.1%)	1		

Alcohol consumption	Yes	11 (50%)	11 (50%)	0.12 (0.4, 0.3)	8.2 (2.17, 31)	0.002^*∗*^
	No	167 (89.3%)	20 (10.7%)	1		

Hemoglobin level	<11 g/dl	16 (53.3%)	14 (46.7%)	8.33 (3.4, 19.9)	8.3 (2.7, 25.6)	<0.001^*∗*^
	≥11 g/dl	162 (90.5%)	17 (9.5%)	1		

MUAC	<23 cm	94 (82.5%)	20 (17.5%)	1.62 (0.7, 3.58)	2.03 (0.72, 5.7)	0.23
	≥23 cm	84 (88.4%)	11 (11.6%)	1		

History of malaria	Yes	19 (76%)	6 (24%)	2.0 (0.73, 5.51)	1.3 (0.34, 4.8)	0.697
	No	159 (86.4%)	25 (13.6%)	1		

1.0 = Referent category, ^*∗*^statistically associated *P* value <0.05, COR = crude odd ratio, AOR = adjusted odd ratio.

## Data Availability

All relevant data are in the manuscript. Additional data used to support the findings of this study are available from the corresponding author upon request.
